# How do medical students engaging in elective courses on acupuncture and homeopathy differ from unselected students? A survey

**DOI:** 10.1186/s12906-017-1653-z

**Published:** 2017-03-09

**Authors:** Alexandra Jocham, Levente Kriston, Pascal O. Berberat, Antonius Schneider, Klaus Linde

**Affiliations:** 1 Institute of General Practice, Klinikum rechts der Isar, Technical University of Munich, Orleansstrasse 47, 81667 Munich, Germany; 2 Department of Medical Psychology, University Medical Center Hamburg-Eppendorf, Hamburg, Germany; 3 TUM Medical Education Center, TUM School of Medicine, Technical University of Munich, Munich, Germany

**Keywords:** Complementary and alternative medicine, Medical education, Attitudes, Personality traits

## Abstract

**Background:**

We aimed to investigate whether students at German medical schools participating in elective courses on acupuncture and homeopathy differ from an unselected group of students regarding attitudes and personality traits.

**Methods:**

Elective courses on acupuncture and homeopathy in the academic half-year 2013/14 all over Germany were identified and participants invited to fill in a questionnaire including nineteen questions on attitudes towards Complementary and Alternative Medicine (CAM), orientation towards science, care and status orientation, and a short validated instrument (Big-Five-Inventory-10) to measure personality traits (extraversion, neuroticism, openness, conscientiousness, and agreeableness). Participants of a mandatory family medicine course at one university served as unselected control group.

**Results:**

Two hundred twenty and 113 students from elective courses on acupuncture and homeopathy, respectively, and 315 control students participated (response rate 93%). Students participating in elective courses had much more positive attitudes towards CAM, somewhat lower science and status orientation, and somewhat higher care orientation than control group students (all *p-*values for three-group comparisons < 0.001). There were no differences between the three groups regarding personality traits with the exception of lower values for agreeableness in controls (*p =* 0.009).

**Conclusions:**

The findings of this study show that attitudes of students participating in elective courses on acupuncture or homeopathy at German medical schools differ to a considerable degree from the attitudes of unselected students.

**Electronic supplementary material:**

The online version of this article (doi:10.1186/s12906-017-1653-z) contains supplementary material, which is available to authorized users.

## Background

The US National Centre of Complementary and Integrative Health pragmatically defines Complementary and Alternative Medicine (CAM) “as a group of diverse medical and health care interventions, practices, products, or disciplines that are not generally considered part of conventional medicine” [1]. From the perspective of skeptical scientists most CAM therapies lack plausibility and a convincing proof of efficacy beyond placebo effects [2]. Yet, the use of CAM therapies is not only widespread in the general population in many industrialized countries [3, 4] but also considerable among physicians, particularly those working in primary care [5, 6]. Given the wide use of CAM it makes sense that medical students learn about the basic principles, risks and state of the evidence on the most important therapies in order to be able to give competent advice to their patients [7]. However, it has been criticized that many CAM courses at medical schools go beyond a critical introduction and actually teach basics how such therapies can be practiced in a rather uncritical manner [8].

In Germany, the use of CAM methods is popular among physicians working in ambulatory care [6] and a number of medical schools offer courses on these subjects [9]. Two therapies which are very widely used are acupuncture and homeopathy [6]. Acupuncture originates from China and involves the insertion of thin needles at specific points of the body to stimulate self-healing. A considerable body of evidence of suggests that acupuncture is likely to be effective in practice for a number of conditions. Yet, correct placement of the needles seems to have limited relevance (e.g. [10]) challenging important tenets of acupuncture. Homeopathy uses highly diluted remedies of agents claimed to cause similar symptoms to those seen in the patient when given in high dose. Many scientists consider homeopathy highly implausible (e.g. [2]) and the interpretation of the available placebo-controlled trials ranges from carefully positive [11] to proving that homeopathy does not work [12].

A number of studies have investigated the attitudes of medical students towards CAM therapies (e.g. [13, 14]), but we are not aware of any studies comparing characteristics and views of students actually engaging in elective courses on such therapies to those of “average” students. Such comparisons are important to understand whether students engaging in CAM are distinct groups. Therefore, we aimed to investigate whether students at German medical schools participating in elective courses on acupuncture and homeopathy differ from an unselected group of students regarding attitudes towards CAM, science, care and status orientation, and personality traits. In addition, we tried to identify classes of students showing similar patterns of attitudes across groups.

## Methods

### Study design, target populations and sampling procedures

The study was a cross-sectional, quantitative, exploratory, anonymous survey. Three groups of students were investigated: an acupuncture, a homeopathy and a control group. The target populations for the acupuncture and homeopathy groups were all medical students participating in an elective course on this subject in the academic half-year 2013/14 at a German medical school. The target population for the control group were all medical students in the same time period in Germany.

To identify all relevant courses on acupuncture (including also courses on traditional Chinese medicine in general) and homeopathy, inquiries were made to faculties and student representatives at all 37 German medical schools. Furthermore, a foundation (Carstens- Stiftung, Essen) and a professional society (German Medical Acupuncture Society) supporting elective courses across Germany provided lists of courses. In summer 2013, contact persons of identified courses were informed about the planned survey and invited to participate, if a course was planned for the half-year 2013/14. Contact persons agreeing to participate then received questionnaires, a short summary for introducing the survey to students, a sheet for documenting the number of questionnaires handed out and collected, and a prepared envelope for sending the material back to the researchers. Participation was voluntary.

The optimal control group would have consisted of a nation-wide random sample of medical students. However, given the limited resources for our study, the lack of a central nation-wide register of medical students and the low likelihood of obtaining high response rates from 37 medical schools, we chose to use a convenience sample. Within a mandatory course on family medicine at the Technical University Munich in November 2013 all participants were invited to fill in the questionnaire. All medical students have to participate in this family medicine course; most do so in their fourth study year (academic half-years 7 or 8), a minority of students at an earlier or later stage.

### Questionnaire

The questionnaire consisted of two modules: module 1 (two pages) for all participants and module 2 (two pages) for students participating in acupuncture and homeopathy courses only. Module 2 included open questions about motives and closed questions about personal experiences, influence of the personal environment and attitudes directly related to the course topic. This paper focuses on methods and findings from module 1; details of the methods and findings for module 2 will be reported elsewhere.

Module 1 had three parts. The first part consisted of 19 statements (see Additional file 1: Table S1 for exact wording) aiming to measure attitudes considered potentially relevant for choosing elective courses or not. Agreement to statements was rated on a 5-point Likert-scale ranging from full agreement (coded as 2) to complete disagreement (coded as −2). Originally, we had assigned the 19 statements to six domains to be summarized in scores. However, confirmatory factor analysis did not completely confirm our postulated factor structure, thus minor modifications for scoring were necessary (see Additional file 2 Digital Content 2 for details). The instrument measured the following scales:

1. “CAM orientation” (a second-order scale consisting of four subscales):
  - “CAM interest” (two statements; internal consistency quantified with Crohnbach’s α = 0.88) addressed the general interest in CAM;
  - “positive attitudes towards acupuncture” (three statements; α = 0.78) the interest in acupuncture, belief in its efficacy and personal experience with it;
  - “positive attitudes towards homeopathy” (three statements; α = 0.80) the interest in homeopathy, belief in its efficacy and, personal experience with it;
  - and “beyond science” (four statements; α = 0.58) agreement to statements indicating an orientation deviating from a scientific view or expressing a critical view of conventional medicine;
2. “Science orientation” (two statements; α = 0.68) attitudes towards a scientific view of medicine;
3. “Care orientation” (three statements; α = 0.61) the willingness to care for others and empathy;
4. “Status orientation” (two statements; α = 0.79) the relevance of status motives for choosing to study medicine.

The second part of module 1 consisted of the Big-Five-Inventory-10 (BFI-10), a short, validated questionnaire to investigate personality traits [15]. The Big-Five is a widely examined theory of five broad dimensions to describe the human personality. The five factors are extraversion, neuroticism, openness to experience, conscientiousness, and agreeableness. The third part of module 1 documented sociodemographic and study- or career-related characteristics.

### Statistics

The study was exploratory (hypothesis-generating). Basic analyses were performed with SPSS 23 software (Armonk, NY). We explored differences between all three groups using the Chi^2^-test (nominal data), the Kruskal-Wallis-test (ordinal data) and ANOVA (summary scales). Pairwise comparisons were done using Fisher’s exact test, the Chi^2^-test, the Mann–Whitney U-test, or the Student t-test. Given the large number of *p-*values calculated these should primarily be interpreted as a reading aid. We did not adjust for multiple testing. The structure of the attitude measure was investigated with confirmatory factor analysis in Mplus 7.2 (see Additional file 3 for details). Participants were classified into homogeneous classes by latent profile analysis. Latent profile analyses (LPA) aim at the grouping of individuals into distinct classes based on response patterns to a defined set of items, so that individuals within a class are more similar than individuals between classes. Usually models with an increasing number of classes are tested and the one with best balance between model fit and parsimony is selected. It is an exploratory procedure with a probabilistic allocation of individuals to classes (rather than setting a deterministic group membership, like, for example, in k-means cluster analysis). We used responses to the 19 attitude statements as input for the LPA. Although previous confirmatory factor analysis revealed a theoretically reasonable factorial structure of the statements, hierarchical factor or scale scores cannot be satisfactorily dealt with in LPA, so that we used the individual items. To express the results, however, we used scale scores due to higher comprehensibility and consistency with rest of the study report. We used a robust maximum likelihood estimator in Mplus 7.2 with 10,000 initial stage random starts, 50 initial stage iterations, and 50 final stage optimizations. Residual correlations between items (i.e., item-correlations within classes) were set to zero. Fit indices did not completely agree regarding the best model. Models with a higher number of classes than three were not significantly better than models with one less class (likelihood ratio tests), but the information criteria suggested that a model with a higher number of classes is necessary. Both the Akaike information criterion and the sample-size adjusted Bayesian information criterion (lower values favorable) decreased continuously with a higher number of classes, while the Bayesian information criterion had a minimum at the model with 6 classes. As the entropy and the size of the smallest class were both favorable of this class, we decided to retain this model for further analyses.

## Results

Information on acupuncture and homeopathy courses could be obtained for 34 (92%) of the 37 medical schools contacted. A total of 18 acupuncture courses were offered at 15 medical schools (41% of 37, assuming that there were no courses at the three medical schools for which no information could be obtained) during the study period. At further four (11%) medical schools acupuncture courses were sometimes offered, but not in the study period. At 10 medical schools (27%) a total of 13 homeopathy courses were offered in the study period (further four (11%) had no current offer). Filled-in questionnaires were obtained from 16 acupuncture (89% of courses offered) and 12 homeopathy courses (92%). A total of 220 questionnaires from acupuncture courses and 113 questionnaires from homeopathy courses could be included in the analysis. In 24 of the 28 participating courses the number of questionnaires handed out was documented; the response rate here was 94%. In the mandatory family medicine course serving as control 315 of 344 (92%) of the registered participants filled in the questionnaire.

Students participating in acupuncture or homeopathy courses tended to be slightly more often female and older in spite of having studied for a slightly shorter time compared to students in the control group (Table 1). Results of the final secondary school examinations were significantly worse in the acupuncture and homeopathy group compared to the control group. Furthermore, students from these groups had more often completed another professional education and had more often a clear idea in which medical area to specialize. In particular, the proportion aiming to specialize in family medicine was higher and the proportion aiming to specialize in surgery was lower than in the control group. A total of 27 (9%) students in the control group reported that they had participated or were currently participating in an elective course on acupuncture (6%) or/and homeopathy (4%).

**Table 1:** Characteristics of participants. Values are absolute frequencies (percentages) or medians (25th and 75th percentile)

Variable (n missing)	Acupuncture (*n =* 220)	Homeopathy (*n =* 113)	Control (*n =* 315)	*p-*value global (pairwise)
Female (9)	161 (73%)	87 (77%)	202 (66%)	.05 (−/*/-)
Age (12)	24 (23, 27)	24 (22, 28)	23 (22, 25)	.005 (**/−/−)
Half-years at medical school (12)	7 (5, 9)	7 (3, 9)	7 (7, 8)	.04 (−/*/*/)
Score secondary school^a^ (28)	1.7 (1.3, 2.3)	1.6 (1.3, 2.0)	1.4 (1.2, 1.6)	<.001 (**/**/-)
Professional training before medical school (9)	69 (31%)	40 (35%)	53 (17%)	<.001 (**/**/-)
Knows planned type of specialization (10) Among those knowing specialization	115 (52%)	63 (56%)	130 (43%)	.015 (*/*/-)
- family medicine	29 (25%)	30 (48%)	19 (15%)	<.001 (−/**/*)
- surgery	13 (11%)	3 (5%)	33 (25%)	.001 (*/*/-)
- internal medicine	13 (11%)	6 (10%)	23 (18%)	.19 (−/−/-)

*p-*values for three-group comparisons from Kruskal-Wallis-tests and Pearson-Chi^2^-tests; *p-*values for pairwise comparisons from Fisher’s exact tests and Mann–Whitney-U-tests: − *p* ≥ .05 ; * *p =* .002 to *p =* .049 ; ** *p* ≤ .001 (order: first position - acupuncture vs. control, second position - homeopathy vs. control; third position - acupuncture vs. homeopathy)

^*a*^scores for final examinations at German secondary schools qualifying for university can vary between 0.7 (best score) and 6 (worst score)

**Fig. 1:**
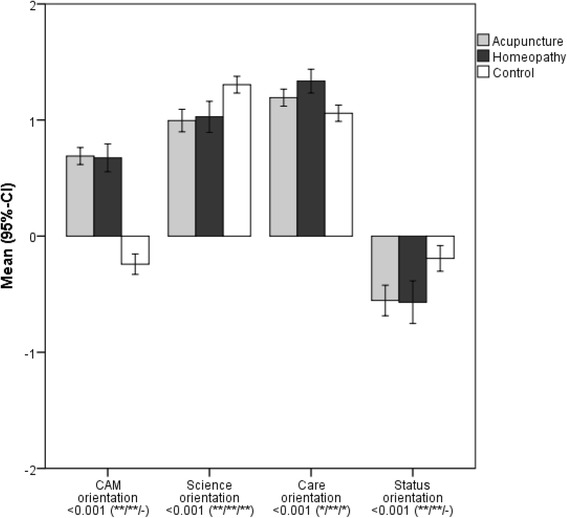
Means (95% confidence intervals) for the four main factors CAM orientation, science orientation, care orientation and status orientation. Higher values indicated stronger agreement. *p-*values for three-group comparisons from ANOVA (significance levels for pairwise comparisons from Student t-tests: − p ≥ 0.05; * *p =*0.002 to *p =*0.049; ** p ≤ 0.001; order: first position - acupuncture vs. control, second position - homeopathy vs. control; third position - acupuncture vs. homeopathy)

There were statistically significant (*p <* 0.001) differences between the groups for the scores for all four main attitude factors (Fig. 1; see Additional file 1 for answers to all individual items and scores). As expected, participants in elective courses on average had positive views towards CAM while control students tended to be neutral to slightly negative on average. The majority of participants considered science important, yet summary scores among acupuncture and homeopathy students were lower than among students in the control group. Care orientation was high in all three groups, but participants of homeopathy courses had the highest scores and control group students the lowest scores. Status motives had a very limited role as reasons for studying medicine among participants of acupuncture and homeopathy courses while they were more important in the control group.

**Fig. 2:**
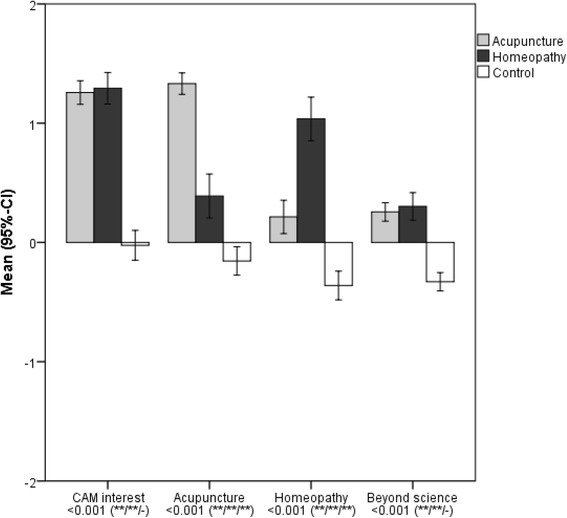
Means (95% confidence intervals) for the four factors CAM interest, acupuncture, homeopathy, and beyond science contributing to the main factor CAM orientation. Higher values indicated stronger agreement. See legend of Fig. 1 for further details

When the four sub-scales contributing to the main factor “CAM orientation” were analysed separately, there were also major differences between the three groups (Fig. 2). The general interest in CAM was similar among acupuncture and homeopathy students and much higher than in the control group. However, the attitude towards the specific complementary therapy chosen as elective course was much more positive than for the complementary therapy not chosen. Agreement to statements indicating an orientation deviating from a scientific view or expressing a critical view of conventional medicine (“beyond science”) was limited also among students participating in the elective courses, yet considerably higher than among control group students.

There were no differences between the three groups regarding personality traits with the exception of lower values for agreeableness in controls (*p =* 0.009; see Additional file 3: Figure S4).

**Fig. 3:**
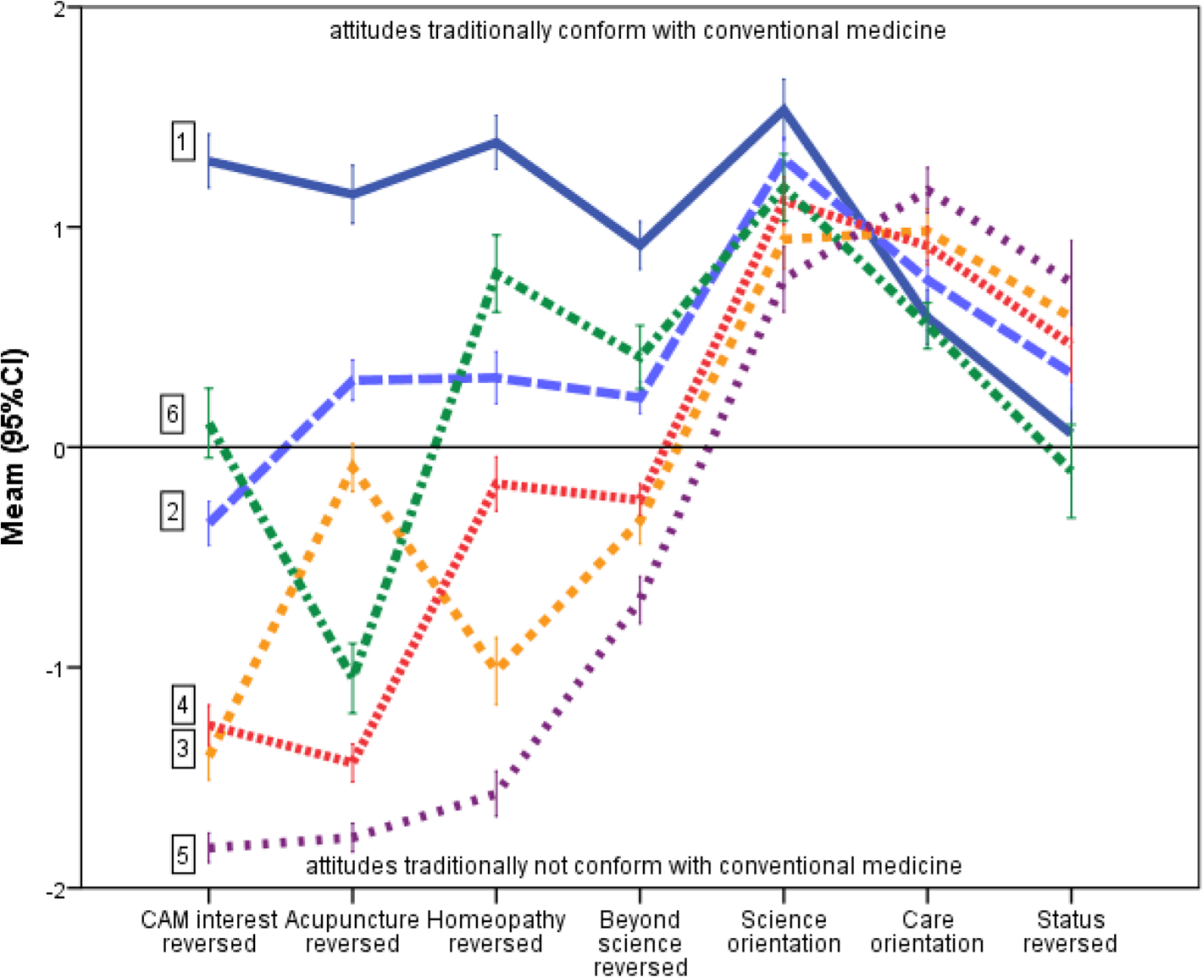
Means (95% confidence intervals) for attitudes according to classes (class number in boxes) identified by latent profile analysis. Note that some scales have been reversed for better graphical separation of classes. The scales CAM interest, Acupuncture, Homeopathy, Beyond Science and Status (orientation) were reversed with positive values now indicating attitudes traditionally conform with conventional medicine academically and negative values attitudes traditionally not conform with conventional medicine

Latent profile analysis suggested six classes of students (see Table 2 and Fig. 3). Absolute differences between classes were very pronounced for CAM attitudes and relatively small for science, care and status orientation. Class 1 (comprising 28% of control group students and only two students in elective courses) was characterized by a rather distinct “anti-CAM orientation”, a strong scientific orientation, and – compared to the other classes - a lower care and a higher status orientation. Class 5 students (24% of acupuncture, 26% of homeopathy and 6% on control group students) can be considered antagonists to class 1 students, as they have a strong CAM, comparably low science, strong care and low status orientation. Class 2 students (comprising 11% of acupuncture, 19% of homeopathy students and 36% of control students) tended to be neutral regarding CAM, had a relatively strong science orientation and were in the middle regarding care and status orientation. Class 3 students (11% of acupuncture, 43% of homeopathy and 9% of control students) had positive attitudes towards CAM in general and homeopathy, but were neutral towards acupuncture, had comparably low science orientation, high care and low status orientation. Instead, among class 4 students (41% of acupuncture, 12% of homeopathy and 8% of control students) attitudes towards acupuncture were quite positive, but neutral regarding general CAM interest, negative towards homeopathy, and these students had a comparably high science orientation. The relative small class 6 (11% of acupuncture, none of homeopathy and 13% of control group students) has a quite unique pattern with rather positive attitudes towards acupuncture and science, but low care and high status orientation. With respect to personality traits class 5 students scored particularly high on openness and conscientiousness, and class 1 students particularly low on agreeableness (results not shown).

**Table 2:** Results of the latent profile analysis per group. Values are absolute frequencies (percentages)

Class – class characteristics (compared to other classes)	Acupuncture (*n =* 220)	Homeopathy (*n =* 113)	Control (*n =* 315)	Total (*n =* 648)
1 – CAM negative, science high, care low, status high	2 (1%)	-	**89 (28%)**	91 (14%)
2 – CAM neutral, science high & care & status moderate	25 (11%)	22 (19%)	**114 (36%**)	161 (25%)
3 – CAM interest strong/Acupuncture moderate/Homeopathy positive, science low, care high, status low	24 (11%)	**49 (43%)**	28 (9%)	101 (16%)
4 – CAM interest strong/Acupuncture positive/Homeopathy neutral, science & care and status moderate	**91 (41%)**	13 (12%)	26 (8%)	130 (20%)
5 – CAM interest strong, science low, care high, status low	**53 (24%)**	**29 (26%)**	18 (6%)	100 (15%)
6 – CAM interest neutral/Acupuncture positive/Homeopathy negative, science moderate, care low, status high	25 (11%)	-	40 (13%)	65 (10%)

Bold data indicate the two most frequent classes per group, respectively

## Discussion

Compared to an unselected control group, students participating in elective courses on acupuncture and homeopathy at German medical schools had on average more positive attitudes towards CAM, considered science less important, had stronger care and lower status orientation, and scored higher on the personality trait agreeableness. Yet, attitudes towards CAM varied much more than science, care and status orientation. If we assume that attitudes of our control group students are broadly representative, it might be expected that about a quarter of German medical students has positive attitudes towards CAM (classes 3, 4 and 5 in Table 2), are at least partly critical about science, and have a high care and a low status motivation. Another quarter (class 1) has strong scientific orientation, is very skeptical about CAM, has lower care and higher status orientation, and scores lower on the personality trait agreeableness. About half of the students (classes 2 and 6) might be expected to be neutral towards CAM, rather scientifically oriented but also to some extent open to heretic views, and have slightly lower care and stronger status orientation than CAM proponents.

To the best of our knowledge, our survey is the first comparing attitudes and personality traits of students participating in elective courses on acupuncture or homeopathy and unselected medical students. The large sample size allowed the reliable detection of relevant differences. The nationwide sampling and the high response rate make it likely that our results give a realistic and representative picture of students participating in such elective courses. An important shortcoming is that we cannot be certain that our control group is representative of German medical students. Compared to many other countries the ranking of universities is much less pronounced in Germany and when choosing a university its reputation is only a secondary criterion [16]. Yet, medical students from the Technical University of Munich compare well with those from other universities regarding the proportion passing the final examination and completing their study in time [17]. Furthermore, for reasons of feasibility our scales for measuring CAM, care and status orientation mostly consist of only two to three items. The individual statements were developed mainly to differentiate the three groups. Our data-driven classification (latent profile analysis) was performed post-hoc with a strictly exploratory approach. Yet, we consider it an interesting part of our work as it allows to postulate some hypotheses regarding patterns of attitudes among (German) medical students.

Multiple studies have shown that medical students in general have positive attitudes towards science (e.g. [18–21]). While the majority of participants of elective CAM courses in our study held positive views about science, too, agreement to the two statements was significantly lower than among participants in the control group. Croatian surveys among students and doctors also found a negative correlation between attitudes towards CAM and science [18, 22]. However, the ratings on our beyond science scale suggest that even many control group students share the view that a purely biomedical scientific approach alone might not be sufficient. For example, 37% agreed to the statement that “conventional medicine does not grasp the patient entirely” and further 37% were uncertain. Also other studies have shown that many medical students are open to more or less non-conventional views [19, 21]. We are not aware of other studies investigating the association of attitudes towards CAM and care or status orientation. Yet, it is an interesting finding that students open to CAM tend to have a somewhat higher care orientation and are more often interested in specializing in family medicine. Competency models for general practice emphasize empathy and perspective taking as key domains [23].

In our study, we did not investigate the contents of the elective courses. Based on what we know from personal contacts we assume that course instructors often are providers of the respective therapy. It seems likely that therapies are taught in a rather “positive” manner and it is unclear whether the courses include a critical discussion of the scientific debates relating to CAM. To what extent and how CAM is taught within the mandatory basic curriculum at German medical schools is unclear but likely to be minimal [9]. The somewhat science-sceptic attitude of many participants in elective CAM courses is a matter of concern. We think our findings provide support for the demand that a critical discussion of the scientific basis of the respective therapy should be a mandatory part in elective CAM courses at medical schools. For the basic curriculum, CAM might be an interesting theme for discussing what is scientific in medicine, what is not, where important limitations are, and where demarcations are professionally inevitable. For example, it has been argued that CAM can serve as a “mirror image” helping to understand scientific reasoning [24]. Discussing CAM examples when teaching evidence-based medicine makes clear how prior beliefs and mechanistic theories shape the interpretation of data [25, 26]. They also make understandable that although placebo *effects* are seen as positive in clinical practice when they are associated with active treatments, placebo *interventions* or interventions without “specific” effects pose major ethical and professional problems [27, 28]. Understanding how a CAM therapy in a specific situation might better address the subjective needs of patients [29] can help to understand fundamental limitations of how modern medical practice approaches the patient [30].

## Conclusion

In conclusion, the findings of our study show that attitudes of students participating in elective courses on acupuncture or homeopathy at German medical schools differ to a considerable degree from the attitudes of unselected students. Further empirical studies using well-validated, comprehensive scales investigating associations between attitudes and personality traits are desirable to investigate whether our findings can be confirmed and whether they apply to other countries.

## Additional files

Additional file 1: Table S1. Agreement to individual statements and summary scales to the 19 questions of module 1, part 1. Values are means (standard deviations = SD) or absolute frequencies (valid percentages). (PDF 138 kb)
Additional file 2: Confirmatory factor analysis of the attitude measure. (PDF 71 kb)
Additional file 3: Figure S4. Means (95% confidence intervals) for Big Five personality traits. (PDF 17 kb)
