# Supplementary material for: How do medical students engaging in elective courses on acupuncture and homeopathy differ from unselected students? A survey
Source: BMC Complement Altern Med. 2017 Mar 9;17:148. doi: 10.1186/s12906-017-1653-z (PMC5343393; doi:10.1186/s12906-017-1653-z)
Supplement: Additional file 1: Table S1. — Agreement to individual statements and summary scales to the 19 questions of module 1, part 1. Values are means (standard deviations = SD) or absolute frequencies (valid percentages). (PDF 138 kb) [file 12906_2017_1653_MOESM1_ESM.pdf]

Table S1

Agreement to individual statements and summary scales to the 19 questions of module 1, part 1. Values are means (standard deviations = SD) or absolute frequencies (valid percentages)

Question (number of missing observations)	Acupuncture (n = 220)	Homeopathy (n = 113)	Control (n = 315)	Total (n = 648)	p-value§
**CAM ORIENTATION**					
**CAM interest**					
I am interested in complementary and alternative medicine (0)					
- mean (SD)	1.3 (0.8)	1.2 (0.8)	-0.1 (1.2)	0.6 (1.2)	
- do not agree at all	0 (0.0%)	0 (0.0%)	41 (13.0%)	41 (6.3%)	<.001
- tend to disagree	9 (4.1%)	3 (2.7%)	79 (25.1%)	91 (14.0%)	(**/**/-)
- uncertain	25 (11.4%)	17 (15.0%)	94 (29.8%)	136 (21.0%)	
- partly agree	78 (35.5%)	42 (37.2%)	67 (21.3%)	187 (28.9%)	
- fully agree	108 (49.1%)	51 (45.1%)	34 (10.8%)	193 (29.8%)	
Basics of complementary and alternative medicine should be taught in medical schools (0)					
- mean (SD)	1.2 (0.8)	1.3 (0.7)	0.1 (1.2)	0.7 (1.2)	
- do not agree at all	1 (0.5%)	0 (0.0%)	39 (12.4%)	40 (6.2%)	<.001
- tend to disagree	6 (2.7%)	2 (1.8%)	66 (21.0%)	74 (11.4%)	(**/**/-)
- uncertain	28 (12.7%)	12 (10.6%)	88 (27.9%)	128 (19.8%)	
- partly agree	93 (42.3%)	44 (38.9%)	82 (26.0%)	219 (33.8%)	
- fully agree	92 (41.8%)	55 (48.7%)	40 (12.7%)	187 (28.9%)	
Scale CAM interest	0.9 (0.6)	0.9 (0.7)	-0.2 (0.9)	0.4 (1.0)	<.001 (**/**/-)
**Acupuncture**					
I am interested in acupuncture (1)					
- mean (SD)	1.7 (0.5)	0.7 (1.0)	0.1 (1.3)	0.7 (1.3)	
- do not agree at all	0 (0.0%)	1 (0.9%)	48 (15.3%)	49 (7.6%)	<.001
- tend to disagree	0 (0.0%)	10 (8.8%)	59 (18.8%)	69 (10.7%)	(**/**/**)
- uncertain	5 (2.3%)	34 (30.1%)	81 (25.8%)	120 (18.5%)	
- partly agree	54 (24.5%)	40 (35.4%)	83 (26.4%)	177 (27.4%)	
- fully agree	161 (73.2%)	28 (24.8%)	43 (13.7%)	232 (35.9%)	
I consider acupuncture an effective therapy (3)					
- mean (SD)	1.4 (0.7)	0.9 (0.9)	0.3 (1.1)	0.8 (1.1)	
- do not agree at all	0 (0.0%)	1 (0.9%)	28 (8.9%)	29 (4.5%)	<.001
- tend to disagree	0 (0.0%)	6 (5.4%)	34 (10.9%)	40 (6.2%)	(**/**/**)
- uncertain	24 (10.9%)	33 (29.5%)	109 (34.8%)	166 (25.7%)	
- partly agree	83 (37.7%)	40 (35.7%)	101 (32.2%)	224 (34.8%)	
- fully agree	113 (51.4%)	32 (28.6%)	41 (13.1%)	186 (28.8%)	
I have personal experiences with acupuncture (1)					
- mean (SD)	0.9 (1.4)	-0.5 (1.7)	-0.8 (1.5)	-0.2 (1.7)	
- do not agree at all	22 (10.0%)	53 (46.9%)	174 (55.4%)	249 (38.5%)	<.001
- tend to disagree	27 (12.3%)	15 (13.3%)	38 (12.1%)	80 (12.4%)	(**/-/**)
- uncertain	19 (8.6%)	5 (4.4%)	20 (6.4%)	44 (6.8%)	
- partly agree	42 (19.1%)	13 (11.5%)	35 (11.1%)	90 (13.9%)	
- fully agree	110 (50.0%)	27 (23.9%)	47 (15.0%)	184 (28.4%)	
Scale acupuncture	1.3 (0.7)	0.4 (1.0)	-0.2 (1.1)	0.4 (1.1)	<.001 (**/**/**)

Additional File 1 to Jocham et al. How do medical students engaging in elective courses on acupuncture and homeopathy differ from unselected students? A survey

Question (number of missing observations)	Acupuncture (n = 220)	Homeopathy (n = 113)	Control (n = 315)	Total (n = 648)	p-value§
**Homeopathy**					
I am interested in homeopathy (1)					
- mean (SD)	0.2 (1.2)	1.3 (0.9)	-0.6 (1.2)	0.0 (1.4)	
- do not agree at all	25 (11.4%)	0 (0.0%)	85 (27.0%)	110 (17.0%)	<.001
- tend to disagree	36 (16.4%)	3 (2.7%)	100 (31.7%)	139 (21.5%)	(**/**/**)
- uncertain	62 (28.2%)	19 (17.0%)	67 (21.3%)	148 (22.9%)	
- partly agree	59 (26.8%)	28 (25.0%)	40 (12.7%)	127 (19.6%)	
- fully agree	38 (17.3%)	62 (55.4%)	23 (7.3%)	123 (19.0%)	
I consider homeopathy an effective therapy (1)					
- mean (SD)	0.1 (1.1)	1.1 (1.0)	-0.5 (1.2)	-0.0 (1.2)	
- do not agree at all	21 (9.5%)	1 (0.9%)	74 (23.6%)	96 (14.8%)	
- tend to disagree	31 (14.1%)	5 (4.4%)	84 (26.7%)	120 (18.6%)	
- uncertain	91 (41.4%)	27 (23.9%)	88 (28.0%)	206 (31.8%)	<.001
- partly agree	54 (24.5%)	32 (28.3%)	53 (16.9%)	139 (21.5%)	(**/**/**)
- fully agree	23 (10.5%)	48 (42.5%)	15 (4.8%)	86 (13.3%)	
I have personal experience with homeopathy (2)					
- mean (SD)	0.3 (1.5)	0.8 (1.5)	-0.0 (1.5)	0.2 (1.5)	
- do not agree at all	41 (18.7%)	16 (14.2%)	83 (26.4%)	140 (21.7%)	<.001
- tend to disagree	30 (13.7%)	16 (14.2%)	43 (13.7%)	89 (13.8%)	(*/**/*)
- uncertain	36 (16.4%)	5 (4.4%)	48 (15.3%)	89 (13.8%)	
- partly agree	45 (20.5%)	17 (15.0%)	74 (23.6%)	136 (21.1%)	
- fully agree	67 (30.6%)	59 (52.2%)	66 (21.0%)	192 (29.7%)	
Scale homeopathy	0.2 (1.1)	1.1 (1.0)	-0.4 (1.1)	0.1 (1.2)	<.001 (**/**/**)
**Beyond science**					
Alternative practitioners are a threat for patients (3) $					
- mean (SD)	-0.5 (1.0)	-0.8 (1.0)	-0.2 (1.1)	-0.4 (1.1)	
- do not agree at all	31 (14.2%)	28 (24.8%)	35 (11.2%)	94 (14.6%)	<.001
- tend to disagree	83 (37.9%)	49 (43.4%)	94 (30.0%)	226 (35.0%)	(*/**/*)
- uncertain	73 (33.3%)	25 (22.1%)	113 (36.1%)	211 (32.7%)	
- partly agree	22 (10.0%)	6 (5.3%)	47 (15.0%)	75 (11.6%)	
- fully agree	10 (4.6%)	5 (4.4%)	24 (7.7%)	39 (6.0%)	
Conventional medicine does not grasp the patient entirely (0)					
- mean (SD)	1.0 (0.9)	0.9 (0.9)	0.2 (1.1)	0.6 (1.1)	
- do not agree at all	2 (0.9%)	1 (0.9%)	19 (6.0%)	22 (3.4%)	<.001
- tend to disagree	15 (6.8%)	5 (4.4%)	64 (20.3%)	84 (13.0%)	(**/**/-)
- uncertain	37 (16.8%)	32 (28.3%)	117 (37.1%)	186 (28.7%)	
- partly agree	102 (46.4%)	40 (35.4%)	79 (25.1%)	221 (34.1%)	
- fully agree	64 (29.1%)	35 (31.0%)	36 (11.4%)	135 (20.8%)	
If a therapy is used for centuries it shows that it is effective (2)					
- mean (SD)	0.4 (0.9)	0.4 (0.9)	-0.3 (1.1)	0.1 (1.0)	
- do not agree at all	8 (3.6%)	5 (4.5%)	52 (16.6%)	65 (10.1%)	<.001
- tend to disagree	26 (11.8%)	8 (7.1%)	75 (23.9%)	109 (16.9%)	(**/**/-)
- uncertain	73 (33.2%)	42 (37.5%)	113 (36.0%)	228 (35.3%)	
- partly agree	94 (42.7%)	47 (42.0%)	67 (21.3%)	208 (32.2%)	
- fully agree	19 (8.6%)	10 (8.9%)	7 (2.2%)	36 (5.6%)	

Additional File 1 to Jocham et al. How do medical students engaging in elective courses on acupuncture and homeopathy differ from unselected students? A survey

Question (number of missing observations)	Acupuncture (n = 220)	Homeopathy (n = 113)	Control (n = 315)	Total (n = 648)	p-value§
I am interested in esotericism (6)					
- mean (SD)	-0.8 (1.0)	-0.9 (1.1)	-1.4 (0.9)	-1.1 (1.0)	
- do not agree at all	63 (28.8%)	42 (37.8%)	182 (58.3%)	287 (44.7%)	<.001
- tend to disagree	81 (37.0%)	40 (36.0%)	88 (28.2%)	209 (32.6%)	(**/**/-)
- uncertain	50 (22.8%)	11 (9.9%)	23 (7.4%)	84 (13.1%)	
- partly agree	19 (8.7%)	14 (12.6%)	15 (4.8%)	48 (7.5%)	
- fully agree	6 (2.7%)	4 (3.6%)	4 (1.3%)	14 (2.2%)	
Scale beyond science	0.3 (0.6)	0.3 (0.6)	0.3 (0.7)	0.0 (0.7)	<0.001 (**/**/-)
Overall scale CAM orientation	0.7 (0.6)	0.7 (0.6)	-0.2 (0.8)	0.2 (0.8)	<0.001 (**/**/-)
**SCIENCE ORIENTATION**					
Science is an important tool to detect the truth (1)					
- mean (SD)	1.2 (0.8)	1.2 (0.7)	1.5 (0.7)	1.3 (0.7)	
- do not agree at all	0 (0.0%)	0 (0.0%)	1 (0.3%)	1 (0.2%)	<.001
- tend to disagree	6 (2.7%)	1 (0.9%)	3 (1.0%)	10 (1.5%)	(**/*/-)
- uncertain	27 (12.3)	18 (16.1%)	19 (6.0%)	64 (9.9%)	
- partly agree	97 (44.1%)	47 (42.0%)	119 (37.8%)	263 (40.6%)	
- fully agree	90 (40.9%)	46 (41.1%)	173 (54.9%)	309 (47.8%)	
Medicine should be firmly grounded on science (2)					
- mean (SD)	0.7 (0.9)	0.8 (0.9)	1.1 (0.8)	0.9 (0.9)	
- do not agree at all	2 (0.9%)	2 (1.8%)	1 (0.3%)	5 (0.8%)	<.001
- tend to disagree	11 (5.0%)	4 (3.5%)	12 (3.8%)	27 (4.2%)	(**/**/-)
- uncertain	69 (31.7%)	34 (30.1%)	47 (14.9%)	150 (23.2%)	
- partly agree	94 (43.1%)	48 (42.5%)	138 (43.8%)	280 (43.3%)	
- fully agree	42 (19.3%)	25 (22.1%)	117 (37.1%)	184 (28.5%)	
Scale scientific orientation	1.0 (0.7)	1.0 (0.7)	1.3 (0.6)	1.1 (0.7)	<.001 (**/**/-)
**CARE ORIENTATION**					
I like to volunteer for other people (1)					
- mean (SD)	1.2 (0.7)	1.4 (0.7)	1.1 (0.8)	1.2 (0.8)	
- do not agree at all	1 (0.5%)	0 (0.0%)	2 (0.6%)	3 (0.5%)	.002
- tend to disagree	3 (1.4%)	1 (0.9%)	10 (3.2%)	14 (2.2%)	(-/*/*)
- uncertain	22 (10.0%)	11 (9.7%)	50 (15.9%)	83 (12.8%)	
- partly agree	110 (50.2%)	43 (38.1%)	145 (46.0%)	298 (46.1%)	
- fully agree	83 (37.9%)	58 (51.3%)	108 (34.3%)	249 (38.5%)	
I am good in putting myself in the position of somebody else (0)					
- mean (SD)	1.1 (0.7)	1.3 (0.6)	1.2 (0.7)	1.2 (0.7)	
- do not agree at all	0 (0.0%)	0 (0.0%)	1 (0.3%)	1 (0.2%)	.329
- tend to disagree	3 (1.4%)	0 (0.0%)	5 (1.6%)	8 (1.2%)	(-/-/-)
- uncertain	26 (11.8%)	13 (11.5%)	38 (12.1%)	77 (11.9%)	
- partly agree	129 (58.6%)	59 (52.2%)	172 (54.6%)	360 (55.6%)	
- fully agree	62 (28.2%)	41 (36.3%)	99 (31.4%)	202 (31.2%)	

Additional File 1 to Jocham et al. How do medical students engaging in elective courses on acupuncture and homeopathy differ from unselected students? A survey

Question (number of missing observations)	Acupuncture (n = 220)	Homeopathy (n = 113)	Control (n = 315)	Total (n = 648)	p-value§
The desire to help other people was an important motivation for me to study medicine (0)					
- mean (SD)	1.2 (0.8)	1.4 (0.8)	0.9 (0.9)	1.1 (0.9)	
- do not agree at all	0 (0.0%)	0 (0.0%)	5 (1.6%)	5 (0.8%)	
- tend to disagree	10 (4.5%)	3 (2.7%)	22 (7.0%)	35 (5.4%)	
- uncertain	29 (13.2%)	11 (9.7%)	52 (16.5%)	92 (14.2%)	<.001
- partly agree	89 (40.5%)	42 (37.2%)	142 (45.1%)	273 (42.1%)	(*/**/-)
- fully agree	92 (41.8%)	57 (50.4%)	94 (29.8%)	243 (37.5%)	
Scale social orientation	1.2 (0.5)	1.3 (0.5)	1.1 (0.6)	1.2 (0.6)	<.001 (*/**/*)
**STATUS ORIENTATION**					
The high social reputation of physicians was a motivation to study medicine (0)					
- mean (SD)	-0.5 (1.1)	-0.5 (1.0)	-0.0 (1.1)	-0.3 (1.1)	
- do not agree at all	39 (17.7%)	21 (18.6%)	33 (10.5%)	93 (14.4%)	<.001
- tend to disagree	79 (35.9%)	39 (34.5%)	72 (22.9%)	190 (29.3%)	(**/**/-)
- uncertain	58 (26.4%)	33 (29.2%)	103 (32.7%)	194 (29.9%)	
- partly agree	38 (17.3%)	16 (14.2%)	90 (28.6%)	144 (22.2%)	
- fully agree	6 (2.7%)	4 (3.5%)	17 (5.4%)	27 (4.2%)	
The high income of physicians was a motivation to study medicine (0)					
- mean (SD)	-0.6 (1.1)	-0.6 (1.0)	-0.3 (1.1)	-0.5 (1.1)	
- do not agree at all	57 (25.9%)	25 (22.1%)	53 (16.8%)	135 (20.8%)	.002
- tend to disagree	65 (29.5%)	43 (38.1%)	94 (29.8%)	202 (31.2%)	(*/*/-)
- uncertain	63 (28.6%)	29 (25.7%)	85 (27.0%)	177 (27.3%)	
- partly agree	30 (13.6%)	12 (10.6%)	69 (21.9%)	111 (17.1%)	
- fully agree	5 (2.3%)	4 (3.5%)	14 (4.4%)	23 (3.5%)	
Scale status orientation	-0.6 (1.0)	-0.6 (1.0)	-0.2 (1.0)	-0.4 (1.0)	<.001 (**/**/-)

P-values from Kruskal-Wallis test (single items) or ANOVA (summary scale) for three-group comparisons, for pairwise comparisons from Mann-Whitney-U or t-test: - p ≥ .05; * p =0.002 to p =0.049; ** p ≤ 0,001 (1. position: acupuncture vs. control, 2. position: homeopathy vs. control; 3. position: acupuncture vs. homeopathy).

$ Item reversely coded for building summary scale
