# Supplementary material for: How do medical students engaging in elective courses on acupuncture and homeopathy differ from unselected students? A survey
Source: BMC Complement Altern Med. 2017 Mar 9;17:148. doi: 10.1186/s12906-017-1653-z (PMC5343393; doi:10.1186/s12906-017-1653-z)
Supplement: Additional file 2: — Confirmatory factor analysis of the attitude measure. (PDF 71 kb) [file 12906_2017_1653_MOESM2_ESM.pdf]

Confirmatory factor analysis of the attitude measure

The confirmatory factor analysis was conducted in Mplus 7.2 using maximum-likelihood estimation and metric indicators in data from 648 participants. Both local and global fit indices were used for model evaluation. Model changes were performed only when necessary with the aim of replicating the theoretically postulated structure as closely as possible.

Originally, we had assigned the 19 statements to six factors or domains to be summarized in scores (sum of ratings/number of items):

1. “CAM interest” (two statements: interest in complementary and alternative medicine in general, basics of complementary and alternative medicine should be taught in medical schools);
2. “Positive attitudes towards acupuncture” (three statements: interest, belief in efficacy, personal experience);
3. “Positive attitudes towards homeopathy” (same three statements as for acupuncture);
4. “Science orientation” (six statements: science is an important instrument to detect the truth, medicine should be based firmly on science, lay practitioner are a health threat, scientific medicine does not grasp the individual person as a whole, long-term use of a treatment is an argument for its effectiveness, interest in esotericism);
5. “Social orientation” (three statements: engagement for other people, desire to help other as motive to study medicine, ability to understand what goes on in other people);
6. “Status orientation” (two statements: high prestige and high income of physicians as motives to study medicine).

The model did not fit the data completely (chi-square test of model fit: chi-square=555.072, df=137, p<.001, normed chi-square=4.05), although most fit indices were acceptable or close to being acceptable (RMSEA=0.069, CFI=0.915, TLI=0.894, SRMR=0.051). Apart from the first two items of the factor “Science orientation” all standardized factor loadings were above 0.40 and statistically significant.

Separating the factor “Science orientation” in two distinct factors (“science orientation” including only the first two statements while the other four statements summarized in a factor) “beyond science” lead to a well-fitting seven-factor model (chi-square=357.994, df=131, p<.001, normed chi-square=2.73, RMSEA=0.052, CFI=0.954, TLI=0.940, SRMR=0.038). However, pairwise intercorrelations among four factors (“CAM interest”, “Positive attitudes towards acupuncture”, “Positive attitudes towards homeopathy”, “Beyond science”) were substantial (ranging from 0.525 to 0.972).

A model assuming that the four strongly intercorrelated factors load on a second-order factor “overall CAM orientation” (hierarchical model) showed good model fit (chi-square=395.968, df=143, p<.001, normed chi-square=2.77, RMSEA=0.052, CFI=0.949, TLI=0.939, SRMR=0.040) with all standardized factor loadings being above 0.45 and statistically significant. All factor intercorrelations in this model were below 0.40. This model was retained for building scores and was used in the study as depicted in the main text.
