# Supplementary material for: How do medical students engaging in elective courses on acupuncture and homeopathy differ from unselected students? A survey
Source: BMC Complement Altern Med. 2017 Mar 9;17:148. doi: 10.1186/s12906-017-1653-z (PMC5343393; doi:10.1186/s12906-017-1653-z)
Supplement: Additional file 3: Figure S4. — Means (95% confidence intervals) for Big Five personality traits. (PDF 17 kb) [file 12906_2017_1653_MOESM3_ESM.pdf]

Additional File 3 to Jocham et al. How do medical students engaging in elective courses on acupuncture and homeopathy differ from unselected students? A survey

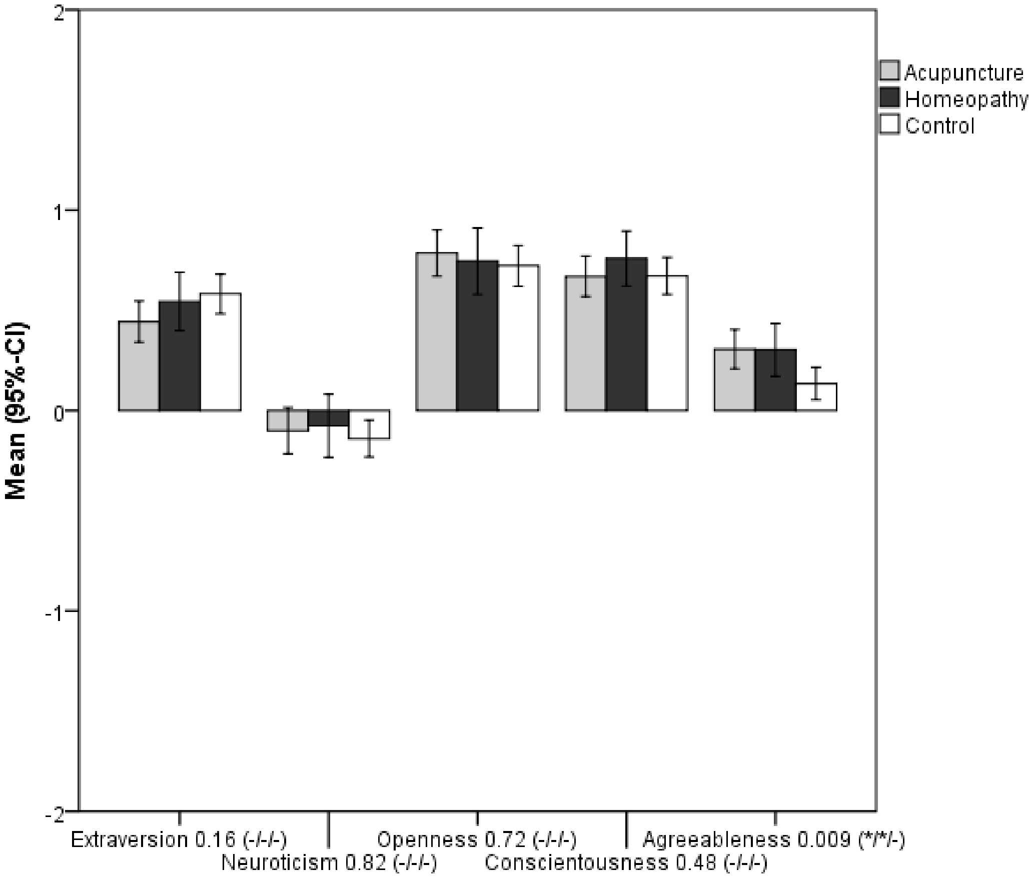

Figure S4

Means (95% confidence intervals) for Big Five personality traits. See legend of Figure 1 for further details.
